# Cost of Community Integrated Prevention Campaign for Malaria, HIV, and Diarrhea in Rural Kenya

**DOI:** 10.1186/1472-6963-11-346

**Published:** 2011-12-21

**Authors:** James G Kahn, Brian Harris, Jonathan H Mermin, Thomas Clasen, Eric Lugada, Mark Grabowksy, Mikkel Vestergaard Frandsen, Navneet Garg

**Affiliations:** 1 Super Models for Global Health, Oakland, California, USA; 2 Philip R. Lee Institute for Health Policy Studies, University of California, San Francisco, 3333 California Street, San Francisco, California, 94118, USA; 3 Coordinating Office for Global Health, Centers for Disease Control and Prevention (CDC)-Kenya, KEMRI Complex, Mbagathi Road off Mbagathi Way, Nairobi, PO Box 606-00621, Kenya; 4 Department of Disease Control, London School of Hygiene and Tropical Medicine, Keppel Street, London, WC1E 7HT, UK; 5 CHF International, P. O. Box 1661 - 00606, Nairobi, Kenya; 6 UN Foundation, 1800 Massachusetts Avenue NW, Suite 400, Washington, DC, 20036, USA; 7 Vestergaard Frandsen SA., Chemin de Messidor 5 - 7, CH - 1006, Lausanne, Switzerland; 8 Vestergaard Asia PVT Ltd., 302 Rectangle One, Saket, New Delhi, 110 017, India

## Abstract

**Background:**

Delivery of community-based prevention services for HIV, malaria, and diarrhea is a major priority and challenge in rural Africa. Integrated delivery campaigns may offer a mechanism to achieve high coverage and efficiency.

**Methods:**

We quantified the resources and costs to implement a large-scale integrated prevention campaign in Lurambi Division, Western Province, Kenya that reached 47,133 individuals (and 83% of eligible adults) in 7 days. The campaign provided HIV testing, condoms, and prevention education materials; a long-lasting insecticide-treated bed net; and a water filter. Data were obtained primarily from logistical and expenditure data maintained by implementing partners. We estimated the projected cost of a Scaled-Up Replication (SUR), assuming reliance on local managers, potential efficiencies of scale, and other adjustments.

**Results:**

The cost per person served was $41.66 for the initial campaign and was projected at $31.98 for the SUR. The SUR cost included 67% for commodities (mainly water filters and bed nets) and 20% for personnel. The SUR projected unit cost per person served, by disease, was $6.27 for malaria (nets and training), $15.80 for diarrhea (filters and training), and $9.91 for HIV (test kits, counseling, condoms, and CD4 testing at each site).

**Conclusions:**

A large-scale, rapidly implemented, integrated health campaign provided services to 80% of a rural Kenyan population with relatively low cost. Scaling up this design may provide similar services to larger populations at lower cost per person.

## Background

Diarrhea, malaria, and HIV together account for one third of total disease burden in Sub-Saharan Africa, as measured in disability adjusted life years (DALYs;[1]). Simple interventions such as the provision of safe drinking water, bed nets, and HIV voluntary counseling and testing, offer the prospect of substantial reductions in disease incidence, morbidity, and mortality[2-4]. The World Health Organization and national governments, including the Government of Kenya, have endorsed principles of universal access to these interventions. Wide access will advance progress toward health targets in the millennium development goals[5].

However, achieving high access is challenging, and has thus far been elusive. In African settings, coverage of voluntary counseling and testing (VCT) ranges from 10% to one-third, with a high proportion of HIV-infected adults (about 80% in Kenya) unaware of HIV status[6]. Surveys in 18 African countries reported in 2008 found that 34% of households owned an insecticide-treated bednet[7]. Use of "improved" water (i.e., piped, a tub well, a protected spring, or rainwater collection) is 49% in rural Kenyan households[8]. Efforts so far have focused mainly on providing ongoing services at health facilities (e.g., for VCT), social marketing (e.g., condoms), and community-based implementation focused on a single disease (e.g., bed net provision and safe water). However, delivering multiple interventions in a single program is increasingly seen as effective and efficient for children; for example, mass immunization campaigns are serving as a platform for providing other child services such as bed nets, deworming medicine and vitamin A[9,10]. However, no comparable strategy exists for adults.

The potential to quickly reach high coverage for multiple prevention interventions - HIV testing, condoms, and counseling; bed nets; and water filters - was recently demonstrated in a community integrated prevention campaign in Western Province, Kenya, implemented by our group[11]. This campaign was designed to address HIV, malaria, and diarrheal disease, to overcome possible resistance to VCT by offering material goods (nets and filters) that offer an additional health incentive for participation, and reduce potential stigma associated with HIV testing. The campaign was based in village centers, where it distributed and instructed on use of bed nets and filters, and offered opt-out VCT. Over one week, more than 80% of the district's estimated 51,178 15-49 year olds attended the campaign. A post-campaign survey indicated commodity use levels of 60% - 90%, depending on the measure used (unpublished data: Grabowsky, M).

Our goals for the present analysis were to (a) document the resources and costs expended by the initial community integrated prevention campaign, and (b) project the cost of scaled-up replication. The latter is based on our observations on operation of the initial campaign and likely efficiencies with routine and larger scale operation, and is supported by data from subsequent community integrated prevention campaign efforts.

## Methods

### Overview of campaign and costing approach

The Integrated Prevention Campaign (IPC) combined prevention interventions for malaria (bed nets), diarrheal disease (filters), and HIV (VCT and condoms). It was implemented in Lurambi District, Western Province, Kenya, over one week (16-22 September, 2008). Recruitment was accomplished with community mobilization.

Activities were conducted simultaneously at 30 village sites, administered as five zones. The campaign used both existing facilities (e.g., churches) and tents set up for the campaign. The IPC reached 47,133 individuals, including 40,749 (80%) of the target age range of 15-49 years old. It provided each participant with HIV VCT; a 'CarePack' containing a long-lasting insecticide treated bed net, a LifeStraw brand water filter, 60 condoms, and educational materials; health education sessions with lecture and demonstrations; and a starter pack of cotrimoxazole (trimethoprim-sulfamethoxazole) prophylaxis for individuals who tested HIV positive.

The IPC was conceptualized by Vestergaard Frandsen (VF) as an extension of past community health campaigns. VF is a privately-owned manufacturer of health products, including the bed nets and filters distributed during the campaign. The IPC was implemented by VF, the Kenyan Ministry of Health (MoH), the Community Housing Foundation (CHF), and EXP Momentum. VF funded the campaign, and the MOH provided campaign personnel, HIV test kits and condoms.

We first determined the cost of the IPC as implemented in the initial demonstration. We employed micro-costing methods, quantifying all provider program costs (excluding participant transportation and time costs), and adopting an "economic" perspective (assigning a fair market value to all resources, whether purchased or donated). We quantified resources and unit costs from planning, logistical, and expenditure reports from VF, CHF, and EXP, clarified by discussions with campaign managers and observation of implementation. We divided costs into traditional budgeting categories (personnel, supplies, services, training, capital, contingency), as well as campaign element (e.g., planning, community mobilization, and implementation).

We then projected the cost of a scale-up strategy, called Scale-Up Replication (SUR). This SUR was first developed theoretically, with key elements confirmed in a subsequent community campaign, as noted below. For the SUR, we assumed 40 sites per geographic area (e.g., a district). The adjustments we made to IPC costs included lower input costs (e.g., Kenyan wages instead of expatriate for on-site management), decreased input intensity (e.g., only one manager per site, justified below), and scale efficiencies (e.g., more counselors per site, and more sites per cycle, leading to broader distribution of fixed costs). The types of adjustments are summarized by campaign element in Table 1. The assumptions came from discussion among the authors, all of whom were directly involved in the IPC execution and several with the subsequent IPC planning. We made no assumptions about increased productivity of front-line staff (e.g., counselors). We examined multiple SUR configurations, with similar results (available on request).

**Table 1:** Cost of Integrated Prevention Campaign (IPC) versus Estimated Scale-Up Replication, per 1,000 individuals (USD)

	IPC		Replication		Reason(s) for difference (in order of magnitude)
**Planning**	$735	1.8%	$96	0.3%	Replication and scale efficiency; increased reliance on Kenyan staffing/wages. *Explanation: *The IPC planning required developing a new campaign, a time-consuming process done by a foreign consultant. In the SUR: 1) Planning activities adapt the initial campaign plan, and then work from one planning template for numerous settings. 2) Staff are Kenyan (81% lower salary), and no foreign travel. The lower cost was confirmed in a subsequent IPC.

**Social Mobilization**	2,141	5.1%	828	2.6%	Scale efficiency; no launch event/concert; Kenyan staffing *Explanation: *Three efficiencies for SUR: 1) The media strategy covers a large area, lowering production and broadcast costs per covered campaign zone. 2) The launch gathering/music event is omitted. 3) Staff are Kenyan, with 81% lower salary. The lower cost was confirmed in subsequent IPC and in a formal bid for a provincial social mobilization.

**Set-up**	2,860	6.9%	975	3.0%	Kenyan staffing; scale & training efficiencies *Explanation: *In IPC: foreigners and a one-time training. In SUR: 1) Staff are Kenyan, with 81% lower salary. 2) Training costs are spread over more campaign participants. 3) Detailed micro-planning is adapted from the IPC.

**Management**	2,428	5.8%	793	2.5%	Kenyan staffing; reduced due to routinization & experienced staff *Explanation: *In IPC: six months' senior manager involvement for a new campaign design, largely foreign. In SUR: 1) Staff are Kenyan, with 81% lower salary. 2) Manager role more time-limited, with routine practices in place. 3) Costs are spread over more campaign participants.

**Implementation**	33,494	80.4%	27,772	86.8%	

***Commodities***	*22,776*		*21,373*		Small scale efficiency. *Explanation: *The manufacturer will cover shipping for filters and nets.

***Personnel***	*8,975*		*5,401*		Kenyan managers reduced due to experienced staff; scale efficiency; Kenyan wages; support staff reductions. *Explanation: *In the initial campaign managers were mainly foreign, and often idle after the first days. Efficiencies in SUR reflect: 1) Fewer managers on-site (1 per site) and off-site, confirmed by field experience in the original and subsequent IPC. 2) Kenyan compensation is 81% below foreign 3) Some non-clinical support staffing is reduced, eg security. 4) Increase from 20 to 25 HIV counselors per site allows more clients per site and thus 20% lower per-client fixed costs. The cost per client of direct service personnel (e.g., HIV counselors) is the same in the IPC and SUR.

***Services***	*968*		*821*		Modest scale efficiency. *Explanation: *Lower domestic transport costs.

**Contingency**	0	0.0%	1,520	4.8%	Added for replication.

**Total**	**41,657**	**100%**	**31,985**	**100%**	

All costs are expressed in 2008 US dollars, using an exchange rate of 68.2 KSh per USD.

### Ethical approvals

This costing study did not require ethical approvals. The overall campaign was deemed exempt from IRB oversight as an operating program in pursuit of formally adopted national health goals, and verbal consent was obtained for HIV testing [11]. The costing relied on campaign implementation records (with no client identifiers) and discussions with implementing managers, methods that are considered exempt from human subjects research oversight.

### Campaign inputs

#### Personnel

HIV counselors providing VCT were the largest group; other staff supported HIV services, other services and site supervision; and zone-or district-level supervisors.

HIV counselors averaged 11.6 per site (range 5-25), serving 20 clients per day each. A further 2-5 individuals supported HIV services: a VCT supervisor, a laboratory supervisor, a person with HIV to assist those who tested positive, and at two sites two CD4 testing staff. Other site personnel included 1-2 site managers, 2 security personnel, 1 registration clerk, 2 health educators to demonstrate proper use of the CarePack contents, 1 distribution clerk, and community volunteers who assisted the clerks and kept the sites clean.

For the SUR we assumed for each site 25 counselors (at the same productivity), CD4 testing staff, 1 health educator, and 1 manager (with Kenyan wages). The reduction to one manager per site is supported by field experience. By day 3 of the Lurambi IPC, one person was managing multiple sites. In a subsequent IPC in nearby Kisii and Kisumu, using refined logistical planning, six sites were managed by two persons (a VF manager resident in Kenya and a Kenyan).

The 5 zones (one per 6 sites) each had 3 zone managers, 1 from VF, CHF, and MOH. A total of 8 storekeepers managed and distributed campaign equipment and supplies at the zone level. District-level staff oversaw the campaign from conceptualization to post-campaign analysis; these were mostly CHF staff, along with personnel from VF and specialists hired for particular tasks. For the SUR, we assumed 2 managers per zone and 1 per district (or other geographic area), and 4 storekeepers, at Kenyan wages.

Our costing of personnel was based on CHF payroll records. For example, counselors were paid $53 per day in wages and per diem, to which overhead and fringe were added. For the 62% of campaign personnel who simultaneously drew MOH salary, that cost was added (representing $13 per day on average). VF personnel were costed according to their average hourly wages and total hours worked.

For the SUR, in summary, we adjusted the personnel mix, unit costs, and quantity. The mix was changed to reflect the altered service configuration (e.g., more counselors per site, to allow a shorter campaign, and thus more per site manager). VF personnel were replaced with Kenyan personnel and pay rates. The total estimate was based on scaling up to serve 10 districts (or similar geographic areas) and 1 million participants. We added 30 personnel for the SUR duration to oversee multi-district operations.

#### Supplies

Commodities, in particular those provided to the participants in the CarePacks, constituted a large portion of IPC costs. CarePacks contained a PermaNet^® ^bednet (unit cost $4.99), a LifeStraw^® ^water filter, 60 condoms (donated by MoH, $2 value), education materials, and a cloth carrying bag. Women received LifeStraw Family (LSF; $20), designed to be mounted in the home and supply clean water for the family; men received LifeStraw Personal (PLSP; $4.75), a compact version designed to be carried while away from the home. HIV test kits donated by MoH had an average cost of $1.40 per client (for the parallel testing protocol used). These unit costs include freight, and we added warehousing and local delivery. For the SUR, we used only slightly lower costs, since production is at scale for bed nets, condoms, and test kits, and nearly at scale for filters, but 6% savings are anticipated due to the company's offer to cover shipping and delivery.

#### Training and support services

Counselors, counselor supervisors, and laboratory supervisors required multi-day training. Their per diem expenses accounted for most of the training costs; these data were derived from CHF records. For the SUR, we budgeted for a single training per employee, who then participate in multiple IPC cycles, rather than one cycle in Lurambi. Thus training represents a lower portion of overall costs.

Services required for IPC included campaign planning; advertising and promotion; travel and lodging; and buses and vans for delivery of staff to the campaign sites. Costing was based on the amounts paid to vendors and appropriate overhead costs. Some costs often considered services (e.g., delivery) were combined with the items they supported (notably CarePacks), and are reflected under supplies. For the SUR, we eliminated foreign travel for VF personnel, and anticipated scale efficiencies for brand-oriented community mobilization, with supportive evidence from planning for a provincial IPC (see Discussion).

### Campaign elements

We divided the campaign cost into 8 elements, reflecting the range of essential functions served by the expenditures. *Planning *included conceptualizing the campaign and specifying a logistics plan. *Social mobilization *comprised meetings with village leaders, and designing and executing media (e.g., posters and radio). *Set-up *involved preparatory work, refinement of logistics procedures, and training. *Management *represented supervisory functions above the sites (e.g., at the zone and district level). *Implementation *was the core of the campaign, the delivery of goods and services to the clients at the site level. *Post-campaign *included wrap-up functions such as completing payroll and storing left over materials. *Monitoring *was the collection and analysis of data on campaign implementation. Finally, for the SUR, we allowed a *contingency *equal to 5% of other expenditures.

We also divided the campaign cost by targeted disease: malaria, diarrhea, and HIV. We first assigned commodities (e.g., bed nets) and dedicated staff time (e.g., HIV counseling) to specific diseases. We then allocated remaining costs (e.g., registration clerks, site and district supervision, tents, and social mobilization) in proportion to the disease-specific spending.

## Results

### Observed Campaign

Costs by campaign element are presented in Table 1, per 1000 individuals served. For the observed IPC, the majority of costs (80.4%) were for the core implementation element (i.e., excluding preparation and follow-up).

The total cost per 1000 individuals served was $41,658. This was comprised two-thirds of commodity costs ($22,776), nearly one-quarter for implementation personnel ($8,975), and the remainder for planning, social mobilization, set-up, and management.

The $41.66 per individual represents $8.32 for malaria, $20.99 for diarrhea, and $12.35 for HIV, including general campaign costs allocated in proportion to disease-specific costs.

### Scaled-up replication (SUR)

For the SUR ("Replication" in Table 1), we projected a greater proportion of costs (86.8%) for the implementation phase. This reflects a lower proportionate reduction in the cost of implementation (17%) than of other campaign elements (such as set-up, mobilization, and planning). Reasons for the difference in cost per 1000 individuals, by campaign element, are noted in the last column of Table 1.

The total cost per 1000 individuals was projected at $31,980. This represents $31.98 per individual, and by disease represents $6.27 for malaria, $15.79 for diarrhea, and $9.91 for HIV. We were able to directly assign 81% of campaign costs to the specific diseases, and allocated the remaining 19% proportionally.

**Figure 1:**
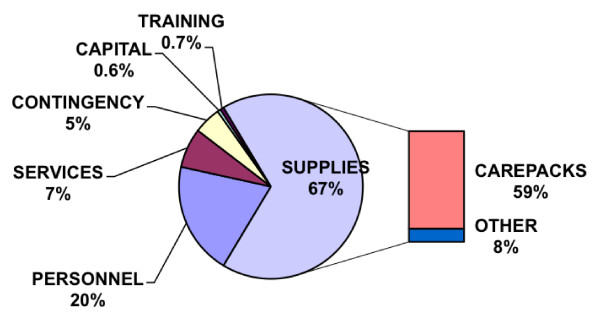
**Expenditure Breakdown for Integrated Prevention, Scaled Up Replication**. Percents >1% rounded to whole numbers.

For the SUR, costs to serve 100,000 individuals, cross-tabulated by campaign element and type of cost, are reported in Table 2. Percent of costs by type is reported in Figure 1. These exhibits emphasize the dominant role of the implementation element in costs, and the major role of supplies (especially care packs), with a substantial subsidiary role for personnel. Of the $31.98 per-person total cost, $28.58 is for actual expenditures (financial costs), and $3.40 is donated (from the MoH: staff salaries, condoms, and HIV test kits).

**Table 2:** Estimated Cost of Integrated Prevention Scale-Up Replication to serve 100,000 individuals (USD)

	Campaign Element								
**Input Type**	**Planning**	**Social Mobilization**	**Setup**	**Management**	**Implementation**	**Post-campaign**	**Monitoring**	**Contingency**	**TOTAL**

**Personnel**	7,381	3,681	56,925	38,389	540,062	458	364		**647,259**

**Training**			20,904						**20,904**

**Services**	2,162	77,011	12,162	32,472	82,135	957			**206,899**

**Supplies**	89	2,126	7,483	4,644	2,137,309		2,054	0	**2,153,706**

**Capital**					17,733				**17,733**

**Contingency**								151,643	**152,325**

**TOTAL**	**9,632**	**82,818**	**97,474**	**75,504**	**2,777,239**	**1,415**	**2,418**	**151,643**	**3,198,826**

Sensitivity analyses suggest that the SUR cost is fairly insensitive to key input uncertainties. The commodity cost has limited uncertainty, due to the already large scale of production; a 10% change would result in a 6.7% change in overall unit cost. Decreasing counselor productivity by 25% (from 20 to 15 clients per day) would increase unit cost by 10% (to $35.23), and increasing it by 25% would decrease unit cost by 6% (to $30.05). Doubling supervisory (managerial) costs would increase overall costs by 4%. If new VCT counselors must be trained, at an estimated USD 1000 each, and each counselor participates in 10 campaigns (seeing 100 clients per campaign), this would increase overall costs by $1 per participant. If we assume resource intensity as in the IPC (e.g., no efficiencies in planning or manager staffing), but Kenyan wages, this yields a per-person cost of $37.

## Discussion

This study documented the cost of a successful community-level integrated prevention campaign for HIV, malaria, and diarrhea in rural Kenya. We used these data to project the cost of achieving further scaled-up coverage of these interventions, with specified anticipated efficiencies in resource use and in unit costs. We project that participants could be provided with a high performance water filter, a long-lasting insecticide-impregnated bed net, HIV counseling and testing, health education regarding the use of these commodities, and 60 condoms at an estimated cost of $31.98 per person.

This estimate is consistent with subsequent experience with IPC implementation in Western Kenya. In a smaller campaign in Kisii and Kisumu (6 sites and 10,206 individuals reached), the total estimated cost per person was $31.08, slightly below the SUR estimate. Planning and set-up costs were $1.19 per person, as compared with $1.07 estimated in SUR. For a planned provincial level implementation, we received a price quote for social mobilization equivalent to $0.45 per person expected to be served by the campaign, lower than our SUR estimate ($0.83).

Compared with prior reports of the cost of providing these services, the integrated campaign approach appears to be efficient. HIV counseling and testing in Africa has had cost estimates ranging widely: $101 in South Africa[12], $26 in Kenya[13], $16 in Kenya[14], $7-11 in South Africa[15], $3-$100 (median $10.50) in Uganda[16], $8-$19 in Uganda[17], and $3-6 in Tanzania[18]. Of these, only one[17] is for programs that achieve high (>90%) community coverage. Our estimate of $9.92 for the SUR integrated campaign is toward the low end of this range, and includes 60 condoms valued at $1.40, as well as CD4 testing for those found to be HIV-positive. A formal comparison across studies is difficult, because varied timing affects both the technology (which has dropped in price), as well as rising prices for consistent inputs such as labor. The door-to-door VCT Uganda cost of $8.29 [17] was for 2007, and thus to be financially comparable to our 2008 estimate would be inflated by 6%^i^, to $8.79.

Long-lasting insecticide-impregnated bed nets have had cost estimates (including delivery) in a narrower range: $6 in Togo in 2004[19], $9-27 including insecticide retreatments in several African settings[20], and $8 in Tanzania in 2006[21]. The cost we estimated for a scaled-up campaign, $6.27, is in the lower end of this range, using a long-lasting bed net[22].

Water filters have been estimated to cost $3 per person-year of use[23]. Transforming the cost of $15.79 per campaign participant (allowing for 2.5 participants per household, 7.73 individuals per household, and two years' benefit) yields an estimated $2.55 per person-year, slightly below the average for filters. Other strategies (e.g., chlorination) are less expensive, but may be less effective, as they require more frequent replenishment of supplies. Solar disinfection, though also low cost and effective, has limited evidence of scalability.

A likely explanation for the low delivery cost is apparent economy of scope in providing all the interventions at once. Seventeen percent of costs were associated with the general campaign infrastructure and general personnel, as compared with 83% for the intervention-specific components (e.g., commodities and training on their use, as well as HIV counseling). If we treat these 17% of costs as fixed, then the incremental costs of adding second and third interventions - i.e., the interventions that "integrate" - are decreased by 17% to $5.20 for malaria, $13.11 for diarrhea, and $8.23 for HIV. Thus, the first intervention incurs the full cost of the general campaign structure, and subsequent interventions require only the incremental costing specific to that disease. In other words, the second and third disease programs build efficiently on the base of the general campaign structure established for the first disease program.

We believe that our findings may apply to other rural areas with a similar mix of disease burden from HIV, diarrhea, and malaria and a similar level of service need for HIV testing and care, clean water, and protection from insect vectors. This might include much of Sub-Saharan Africa and many parts of Asia.

Our analysis has important limitations. First, our estimate of the cost of scaled-up replication (SUR) is based on our best assessment of potential reductions in resource intensity and unit costs, albeit supported by subsequent IPC activities. We could not find a validated method for this analysis. As shown in the sensitivity analyses, key uncertainties are associated with a 4-10% variation in results. Empirical verification with a scaled-up implementation is imperative. This is especially true for different country settings, where political, social, and health care contexts may affect IPC logistics and participation.

Second, we examined the cost of using specific commodities, such as the LifeStraw^® ^water filter. Filters were an integral component of social mobilization for the campaign, serving as an incentive to achieve high VCT coverage. We cannot predict participation with a different approach.

Third, our analysis did not examine the effect of the IPC. We have separate publications under review on commodity uptake and use, and on cost-effectiveness, as well as two encouraging qualitative assessments of reactions to IPC participation [24,25]. A post-campaign survey found commodity use levels of 60% - 90% (unpublished data, Grabowksy, M). Sustained use (e.g., 2-3 years) is essential to benefit, though we note that studies of the magnitude of health effects of commodity distribution are typically conducted at the community level, not for individuals with confirmed use. Commodity use should be encouraged by the substantial on-site health education component. Further, the Ministry of Health was a key partner in implementation, and referrals for HIV care were coordinated with local providers.

Fourth, it is possible that a more efficient, larger campaign might compromise quality. Key features of campaign quality include: ability to attract participants; acceptability of the participant experience; effective training in the use of nets and filters; HIV counseling and testing up to usual standards; and commodity use in the community. However, we believe that there is reason to be optimistic. As noted above, service delivery staffing in the SUR is at levels used in the campaign. Manager staffing is above levels found adequate in the latter parts of the campaign and in a subsequent smaller campaign. Ultimately, both efficiency and quality must be directly assessed in a campaign implemented on a larger scale, e.g., provincial.

Various managerial strategies may be employed to achieve efficient scale-up. A modular approach, in which several geographically defined population groups are targeted each week, appears reasonable to us, and is reflected in our SUR analysis. This approach builds on the initial implementation, which was in effect one module. The number and size of modules can be adjusted according to the availability of HIV testing counselors (likely the limiting resource) and the desired speed of large-scale implementation.

Financial incentives are another tool. For example, productivity-based payment (a fixed amount per campaign participant) may encourage continued or increased efficiency. Quality and financial controls may be especially important under such a system. For example, if the average counselor time per individual receiving VCT is shortened, direct supervision and counseling records must assure that key risk reduction messages are delivered and that HIV-infected individuals, who appear to offer the best opportunity for significant risk reduction, are fully and effectively counseled.

## Conclusion

The economic value of an integrated community campaign appears substantial: the ability to achieve high levels of coverage with economic efficiency equal or potentially superior to non-integrated (vertical) programs, without having to organize multiple programs. If the fully scaled up programs accomplish the intended coverage and efficiency, the potential public health benefits may be large.

## Competing interests

JGK and BH were contracted to conduct this analysis by Vestergaard Frandsen, which manufactures the bed nets and water filters used in the campaign; JGK retained full control of publication and editorial decisions. MVF and NG are owner and senior executive, respectively, for VF, with competing interest forms available on request. EL and TC were paid by VF for other work related to this campaign. There are no other competing interests, financial or nonfinancial.

## Authors' contributions

JGK designed the study, oversaw data collection, guided analyses, and drafted the manuscript. BH assisted in study design, designed and implemented data collection, conducted analyses, and edited the manuscript. JM advised on study design and participated in manuscript revision. TC advised on study design and edited the manuscript. EL assisted with data collection, advised on analysis, and edited. MG advised on study design and edited. MVF conceived the study and edited the manuscript. NG conceived the study, participated in study design, assisted with data collection and analysis, and edited and drafted the manuscript. All authors read and approved the final manuscript.

## Pre-publication history

The pre-publication history for this paper can be accessed here:

http://www.biomedcentral.com/1472-6963/11/346/prepub

## Endnotes

^i ^http://www.indexmundi.com/uganda/inflation_rate_%28consumer_prices%29.html
